# GFAPδ Expression in Glia of the Developmental and Adolescent Mouse Brain

**DOI:** 10.1371/journal.pone.0052659

**Published:** 2012-12-21

**Authors:** Carlyn Mamber, Willem Kamphuis, Nina L. Haring, Nuzrat Peprah, Jinte Middeldorp, Elly M. Hol

**Affiliations:** 1 Department of Astrocyte Biology & Neurodegeneration, Netherlands Institute for Neuroscience - an Institute of the Royal Netherlands Academy of Arts and Sciences (KNAW), Amsterdam, The Netherlands; 2 Department of Neurology and Neurological Sciences, Stanford University School of Medicine, Stanford, California, United States of America; 3 Swammerdam Institute for Life Sciences, Center for Neuroscience, University of Amsterdam, Amsterdam, The Netherlands; Nathan Kline Institute and New York University School of Medicine, United States of America

## Abstract

Glial fibrillary acidic protein (GFAP) is the major intermediate filament (IF) protein in astrocytes. In the human brain, GFAP isoforms have unique expression patterns, which indicate that they play distinct functional roles. One isoform, GFAPδ, is expressed by proliferative radial glia in the developing human brain. In the adult human, GFAPδ is a marker for neural stem cells. However, it is unknown whether GFAPδ marks the same population of radial glia and astrocytes in the developing mouse brain as it does in the developing human brain. This study characterizes the expression pattern of GFAPδ throughout mouse embryogenesis and into adolescence. Gfapδ transcripts are expressed from E12, but immunohistochemistry shows GFAPδ staining only from E18. This finding suggests a translational uncoupling. GFAPδ expression increases from E18 to P5 and then decreases until its expression plateaus around P25. During development, GFAPδ is expressed by radial glia, as denoted by the co-expression of markers like vimentin and nestin. GFAPδ is also expressed in other astrocytic populations during development. A similar pattern is observed in the adolescent mouse, where GFAPδ marks both neural stem cells and mature astrocytes. Interestingly, the Gfapδ/Gfapα transcript ratio remains stable throughout development as well as in primary astrocyte and neurosphere cultures. These data suggest that all astroglia cells in the developing and adolescent mouse brain express GFAPδ, regardless of their neurogenic capabilities. GFAPδ may be an integral component of all mouse astrocytes, but it is not a specific neural stem cell marker in mice as it is in humans.

## Introduction

Glial fibrillary acidic protein (GFAP) is a type III intermediate filament protein (IF; for review see [1], [2]). IFs play important roles in cytomechanics and cell signaling [3]–[5]. GFAP is one of the IFs expressed by radial glia, adult astrocytes, and neural stem cells [6]–[9]. GFAP has several splice variants. The canonical isoform, GFAPα, contains nine exons and is the most abundantly expressed isoform in the human and mouse central nervous system [2], [10]. Another isoform, GFAPδ, differs from GFAPα in its unique carboxy-terminus, created by the replacement of exons 8 and 9 with exon 7+/7a [11], [12]. This structure renders the assembly of GFAPδ compromised, in that it is unable to form filaments by itself. For proper filament formation, another type III IF protein, such as GFAPα, is required [13], [14]. The ratio of GFAPα and GFAPδ has shown to be important factor in IF network formation [10], [14]. Transfection of IF free cells with a GFAPα/GFAPδ ratio of 3∶1 already results in an aberrant condensed IF network. Ratios such as 1∶1 and 1∶3 result in improper filament formation [10]. This aberrant network formation may have functional consequences. GFAPδ itself has already been shown to be involved with the γ-secretase complex via its specific interaction with presenilin [15]. The γ-secretase complex is a crucial mediator of Notch signaling and therefor important for stem cell biology. It is via this pathway that GFAPδ is thought to be linked with neurogenesis.

Humans begin to express GFAPδ at the same time pan-GFAP immunoreactivity is observed, around gestational week 13. This GFAPδ expression is specifically found in radial glia, as denoted by co-expression of radial glial markers such as vimentin and nestin [16]. Radial glia are a type of precursor cell located in the ventricular zone (VZ) and in the medial pallium (MPall), the developing hippocampal formation [17]–[19]. Later in development, the VZ becomes the adult subventricular zone (SVZ) and the MPall transforms into the adult hippocampus. Radial glia are a heterogeneous population of cells that are able to self-renew and produce neurons as well as glia [20], [21]. The production of neurons and glia is temporally dependent, with the peak of neurogenesis being around embryonic day 15 (E15) and the peak of radial glia-dependent gliogenesis around postnatal day 0 (P0) in rodents [20], [22], [23]. Interestingly, a second wave of gliogenesis takes place locally in the cortex during the first postnatal week of life [24]. As embryonic stages progress into postnatal ages, radial glia undergo direct transformation into astrocytes [25], [26] but a small population of these astrocytes is thought to reside as neural stem cells in the adult brain.

GFAP expression in the developing mouse brain follows the basic progression of developing radial glia and astrocytes. Gfap transcripts can first be detected in the mouse brain between E9.5 and E11 [27], corresponding with the appearance of the first radial glia [28]. To note, there is a difference in GFAP expression timing between human and mouse. GFAP is first seen much earlier in mouse (corresponding to around 4.3–6.1 human gestational weeks) than in humans (13 gestational weeks [29]). GFAP is first expressed by radial glia around the telencephalic VZ, in the MPall, and fimbria (fi) [7], [30]. Levels of Gfap mRNA at these early timepoints are very low, but as development progresses and gliogenesis commences, Gfap transcripts become abundantly expressed. After gliogenesis, Gfap levels plateau in the adult brain [30], [31].

Neurogenesis continues in the adult brain, however it is much more restricted [9]. There are two major neurogenic niches of the adult brain, the SVZ located along the lateral wall of the lateral ventricle, and the subgranular zone (SGZ) in the hippocampus [32]–[36]. These areas are present in both humans and rodents [37], [38]. Interestingly, GFAPδ is highly expressed in the adult human SVZ [14]. It co-labels with stem cells markers such as sex-determining region Y-box 2 (Sox2) and nestin, as well as the cell division markers minichromosome maintenance complex component 2 (MCM2) and proliferating nuclear antigen (PCNA). Primary adult human neurosphere cultures also express GFAPδ along with nestin and the cell division marker Ki67 [39]. This population of GFAPδ cells in the SVZ has been shown to be the quiescent neural stem cells of the adult human brain [39], [40]. Notably, GFAPδ is expressed in other human brain regions such as the olfactory bulb, rostral migratory stream, and glia limitans. In the adult mouse brain, immunostainings have shown that GFAPδ is expressed in most astrocytes throughout the brain that express detectable levels of GFAPα, including astrocytes in the SVZ. Moreover, transcript levels of Gfapδ in relation to the canonical isoform Gfapα (Gfapδ/Gfapα transcript ratio) remain constant amongst neurogenic and non-neurogenic areas [10], suggesting that GFAPδ is not a specific neural stem cell marker in the adult mouse brain.

This study takes a closer look at mouse GFAPδ in relation to development and early adulthood. The expression of GFAPδ in the developing mouse brain was examined using immunohistochemistry (IHC) and quantitative real time PCR (qPCR). Surprisingly though Gfapδ transcripts were detectable from E12, GFAPδ protein was only found from E18. For IHC experiments, special attention was paid to the developing SVZ and hippocampus from E12 to P10, as these areas house radial glia during development and maintain their neurogenic capacity throughout life. Unlike the situation in the human brain, GFAPδ did not demarcate a specific population of cells. All cells that expressed GFAP throughout the brain (indicated by a pan-GFAP antibody), also expressed GFAPδ. *In vitro*, GFAPδ was present in both primary mouse astrocyte and neurosphere cultures. These data indicate that GFAPδ may hold a different function in the mouse, as it is expressed in similar levels throughout all types of astrocytes in the developing and adolescent mouse brain.

## Materials and Methods

### Animals and Tissue Preservation

All experiments were carried out under the approval of the Animal Experimentation Committee of the Royal Netherlands Academy of Arts and Sciences (KNAW) with accordance to the European Community Council directive of November 24, 1986 (86/609/EEC). All efforts were made in order to minimize both the number and suffering of the animals involved in the current study.

For primary cultures, C57BL/6 pups between P0 and P3 were cooled on ice. They were decapitated and their heads were kept in cold DMEM (Life Technologies) until astrocyte or neural stem cell isolation.

For GFAP isoform transcript profiling during development, two nests of E12, E15, E18, and postnatal day 0 (P0) were used. Plug date was defined as E0. Mothers were sacrificed with an overdose of pentobarbital (0.40 ml/100 g) followed by cervical dislocation. Embryos were removed from the uterus and decapitated. Their brains were rapidly dissected and put directly into TRIsure (Bioline) or TRIzol (Invitrogen) for RNA isolation. P0 pups were first cooled on ice and then decapitated. Their brains were also quickly removed from the skull and homogenized in TRIsure. For immunohistochemistry (IHC), embryos were first cooled on ice and snap frozen in their entirety. For P0 and P5, pups were cooled on ice, decapitated, and then their heads were snap frozen. For later ages (P10 and P25), mice were given an intraperitoneal (i.p.) overdose of pentobarbital (0.15 ml/100 g), then perfused with saline followed by 4% paraformaldehyde (PFA) in phosphate buffered saline (PBS; pH 7.4), followed by rapid brain dissection. After cryoprotection with 20% sucrose-PBS, their brains were snap frozen. All IHC tissue was stored at −80°C until further use. A cryostat (Leica CM3050) was used to cut 10 µm sagittal sections. These sections were then mounted on Superfrost Plus slides (Menzel-Gläser), dried and stored at −20°C.

### Primary Cell Isolation

For primary cell isolation, pups between P0 and P3 were used. After removal of the olfactory bulbs and cerebellum, the brain was chopped into small pieces and incubated with 2.5% trypsin (Invitrogen) for 5 min at 37°C. Deoxyribonuclease I from bovine pancreas (8 µl/ml, DNaseI; Sigma-Aldrich) was then added and cells were incubated at 37°C for an additional 5 min. A Pasteur pipette was used for the final dissociation step. DMEM with 10% Fetal Bovine Serum (FBS; Invitrogen) was added to the tube, cells were then spun at 1200 rpm for 10 min. The pellet was washed with 0.25% fungizone (Invitrogen) in DMEM. Cells were spun again, resuspended and then plated. For neural stem cell cultures, cells were plated in 6 well dishes (Greiner bio-one) with DMEM:F10 (Invitrogen) plus 1% penicillin streptomycin (penstrep; Invitrogen), 1% glutamine (Lonza), 1% N2 (Invitrogen), 20 ng/ml EGF (Peprotech/Tebu-bio), and 10 ng/ml bFGF (Peprotech/Tebu-bio). Growth factors were added twice a week. Astrocytes were plated in poly-L-lysine (PLL; Sigma-Aldrich) coated T75 flasks in DMEM:F10 plus 1% penstrep, 10% FBS, 0.25% fungizone. Cultures were purified approximately one week after isolation. Astrocyte cultures were taped onto a Unimax 2010 shaker (Heidolph, Schwabach, Germany) inside an incubator and shaken at 240 rpm for 2 hours. The supernatant was removed and cells were given fresh medium [41]. This procedure resulted in a mixed glia culture (predominantly microglia and astrocytes) where astrocytes represented around 80% of all cells based on immunohistological analysis of astrocyte markers such as GFAP and Vimentin.

### RNA Isolation and cDNA Synthesis

After homogenization in TRIsure or Trizol, chloroform was added and samples were centrifuged. The aqueous phase was removed and mixed with an equal amount of isopropanol and 1 µl glycogen (Roche). RNA was allowed to precipitate for at least 2 days at −20°C. Samples were then centrifuged, the pellet washed twice with 70% ethanol, and air-dried. RNA pellets were resuspended with sterile MilliQ. RNA concentrations were determined using a Nanodrop (ND-1000; ThermoScientific, Wilmington, DE, USA). A fixed amount of RNA (250 ng) was used for cDNA synthesis carried out under manufacturer’s protocol using the Quantitect Reverse Transcription Kit (Qiagen).

### Quantative Real Time PCR (qPCR)

qPCR was performed as described previously [42]. Three different primer pairs were used to detect Gfapα, Gfapδ, and pan-Gfap (representing all Gfap isoforms except Gfap ? ; see Table 1). Housekeeping genes were used to normalize all data, these were as follows: Glyceraldehyde-3-phosphate dehydrogenase (Gapdh; TGCACCACCAACTGCTTAGC/GGCATGGACTGTGGTCATGA) and β-actin (Actb; GCTCCTCCTGAGCGCAAG/CATCTGCTGGAAGGTGGACA). Normalized expression values were calculated as described previously [43].

**Table 1:** Specific primers for different Gfap isoforms.

Transcript	Primer	Sequence	Primerlocation
pan-Gfap	Forward	5′-AAGCCAAGCACGAAGCTAACGA-3′	Exon 1
	Reverse	5′-TTGAGGCTTTGGCCCTCC-3′	Exon 2/3
Gfapα	Forward	5′-GGAGATGCGGGATGGTGAG-3′	Exon 8
	Reverse	5′-ACCACGTCCTTGTGCTCCTG-3′	Intron 8/9 andExon 9
Gfapδ	Forward	5′-TCTCCAACCTCCAGATCCGA-3′	Exon 7
	Reverse	5′-TGACTTTTT///GGCCTTCCCCT-3′	Intron 7/8

Different primer pairs were used to specifically investigate Gfap isoform transcript level expression [10].

### Immunohistochemistry (IHC) and Microscopy

Immunohistochemistry was carried out as described previously [42]. The following primary antibodies were used: rabbit anti pan-GFAP (1∶2000; DAKO), mouse anti pan-GFAP (1∶4000; G3893 clone G-A-5, Sigma), rabbit anti GFAPδ (1∶500; Bleeding date: 10.12.2003) [10], goat anti GFAP C-19 (specific for the GFAPα C-terminus; 1∶500; sc-617, SantaCruz), chicken anti-Nestin (1∶1000; NB100–1604, Novus Biologicals), chicken anti-Vimentin (1∶4000; AB5733, Chemicon), goat anti-MCM2 (1∶1500; sc-9839, Santa Cruz), and rabbit anti-pHH3 Cy5 conjugated (1∶200; 9716, Cell Signaling). All secondary antibodies were used at a final concentration of 1∶1400. The secondary antibodies were as follows: Donkey anti-Rabbit Cy3, Donkey anti-Mouse Alexa488, Donkey anti Mouse DyLight488, Donkey anti-Mouse Cy5, Donkey anti-Goat Alexa488, Donkey anti-Goat Dylight649, Donkey anti-Chicken DyLight488, and Donkey anti-Chicken Cy5 (Jackson ImmunoResearch). The stained sections were analyzed using either a Zeiss Axioplan Neofluar fluorescence microscope (Zeiss, Göttingen, Germany) or a Leica SP5 confocal DMI6000 microscope (Leica, Wetzlar, Germany).

For immunostaining of primary astrocyte cultures, astrocytes were plated on poly-L-lysine (PLL) coated glass coverslips. Cells were fixed with 4% PFA in PBS (pH 7.4) for 10 minutes and washed twice with PBS. Cells were blocked with supermix (50 mM Tris, 154 mM NaCl, 0.25% Gelatin, 0.5% Triton-X-100 in H_2_O, pH 7.4; sumi) and incubated with primary antibodies diluted in sumi overnight at 4°C. The next day, cells were washed three times with PBS (pH 7.4) and incubated with secondary antibody diluted in supermix for 1 hour at room temperature.

## Results

### The GFAPδ Protein is Detected Later in Development than other GFAP Isoforms

To localize where and when GFAPδ is first expressed, immunohistochemistry was performed on embryonic (E) and early postnatal (P) mice. In order to study the contribution of GFAPδ expression to the total GFAP expression level, the GFAPδ staining patterns were compared to that of the DAKO GFAP antibody (pan-GFAP). Particular focus was paid to the ventricular zone (VZ) and developing hippocampus (medial pallium; MPall), as these regions contain radial glia. GFAPδ localization throughout various brain regions over all ages studied is summarized in Table 2.

**Table 2:** Progression of GFAPδ expression throughout the developing mouse brain.

	Cerebellum	Cortex	Fimbria	GliaLimitans	Hippocampus	Muller Glia	Olfactory Bulb	Optic Nerve	Striatum	SVZ
E18	−	−	+++	+	+	−	−	++	−	++
P0	−	+	+++	++	+	−	−	+++	−	++
P5	+	+	+++	++	+	+	+	+++	+	++
P10	++	+	++	++	+	n.d.	+	n.d.	+	++
P25	++	+	+	++	+	n.d.	+	n.d.	+	++
P45	++	+	+	++	+	n.d.	+	n.d.	+	++

GFAPδ expression was followed in major brain regions from E18 to P45. Strikingly, GFAPδ immunoreactivity increases in some brain regions, such as the cerebellum, cortex, olfactory bulb and striatum, as the animal ages. While other brain regions, such as the fimbria, gradually downregulate GFAPδ expression. Legend: −not expressed; **+** lowly expressed; **++** moderately expressed; **+++** highly expressed; **n.d.** not determined. For P10 to P45, the eyes and optic nerve were not preserved, as only the brains of these animals were processed for analysis.

**Figure 1:**
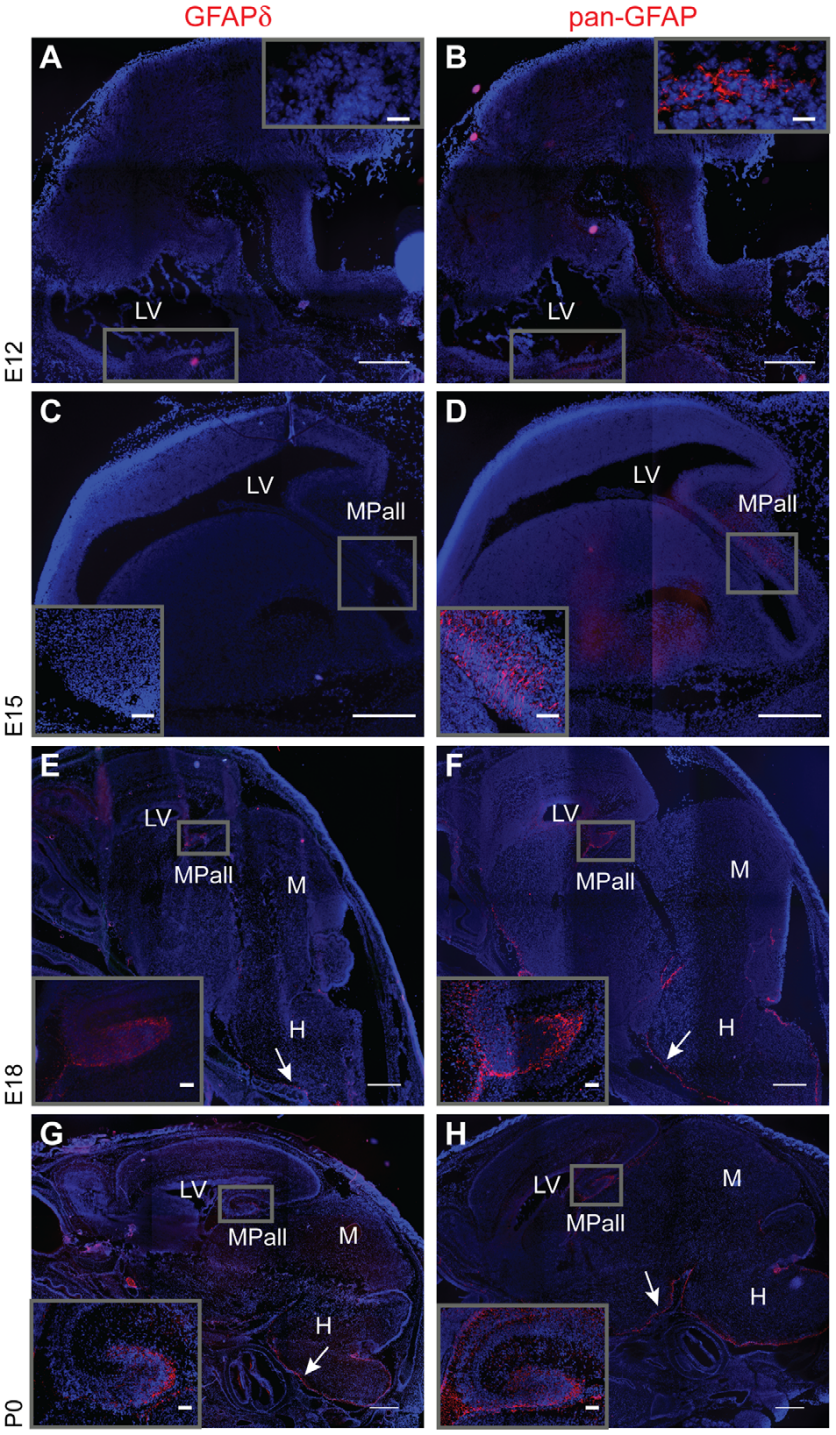
GFAP expression during mouse development. Serial sections of embryonic brains were stained with an antibody that recognizes most GFAP isoforms (pan-GFAP; B, D, F, H) or a specific GFAPδ antibody (A, C, E, G). GFAP first appeared at E12 (B) whereas GFAPδ was absent at E12 and E15 (A and C). At E18 and P0, GFAPδ expression mimicked the expression profile of GFAP isoforms (E–H). Arrows indicate the glia limitans. All pictures were recorded at comparable settings and contrast was enhanced to an identical degree. Boxes located in the upper right hand corner of A and B represent enlargements of the E12 ventricular zone. Insets located in the lower left corner of C–H show an enlarged image of the MPall at E15 through P0. Abbreviations: H: hindbrain, LV: lateral ventricle, MPall: medial pallium, M: midbrain. Scale bars = 500 *µ*m.

Pan-GFAP immunoreactivity was first observed at E12, while GFAPδ immunoreactivity was completely absent (Fig. 1A,B). As the GFAPδ antibody is specific [10], this lack of GFAPδ immunoreactivity at E12 indicates that there is little to no GFAPδ protein expression. Pan-GFAP expression was weak and confined to parenchyma directly surrounding ventricular areas. At E15, the pan-GFAP immunoreactivity spread and was observed mostly in the VZ and the MPall. Yet still, GFAPδ could not be detected (Fig. 1C,D). At E18, the pan-GFAP immunoreactivity grew, infiltrating more area of the VZ and MPall as well as the glia limitans, and GFAPδ was observed for the first time (Fig. 1E,F). The GFAPδ staining was mainly observed in the VZ, MPall, and along the pial surface near the hindbrain (Fig. 1G). At P0, pan-GFAP immunoreactivity continued to demarcate a greater population of cells, while the expression of GFAPδ did not appear to shift in any way (Fig. 1G,H).

**Figure 2:**
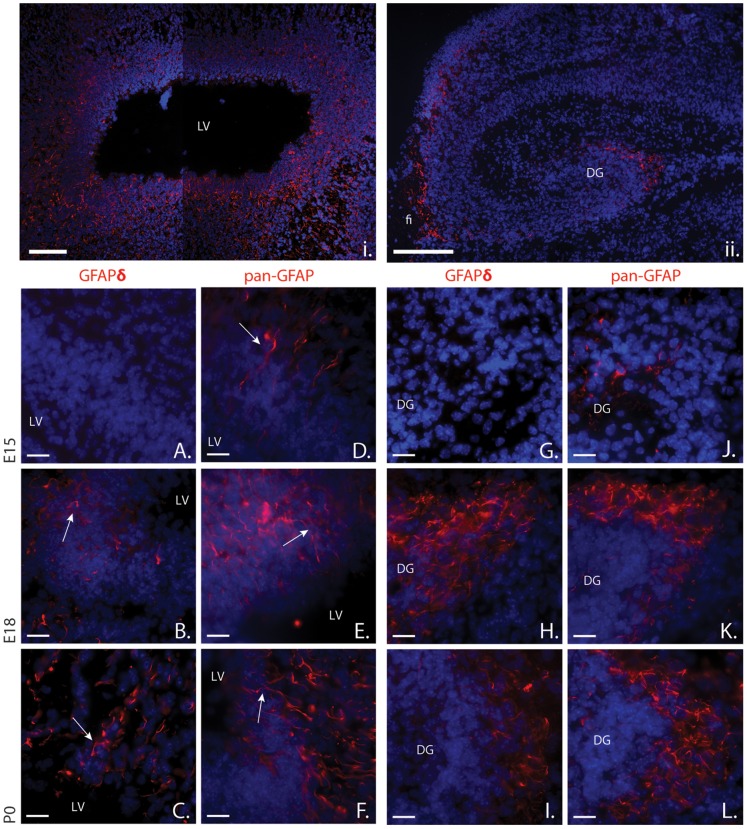
GFAP expression in the ventricular zone and medial pallium. i. At E18, GFAP isoforms can be strongly detected all around the ventricle. Throughout development, cells that express GFAP isoforms have a clear bipolar morphology (arrows, D–F). GFAPδ is absent at E15 (A) and begins to be expressed at E18 in a similar population of cells as other GFAP isoforms (arrows, B–C). ii. At E18, most cells expressing GFAP isoforms in the MPall are clustered in the DG and the fimbria. In the DG, expression of GFAP isoforms increases over time (J–L). Like in the VZ, GFAPδ is first expressed in the MPall at E18 (E). GFAPδ displays a similar expression intensity at E18 and P0 (I). i. and ii. are tiled images which give a detailed overview of their corresponding structures. Abbreviations: DG: dentate gyrus, fi: fimbria, LV: lateral ventricle. Scale bars = 100 *µ*m (i and ii) or 20 *µ*m (A–I).

Pan-GFAP immunoreactivity was observed to surround the lateral ventricle (Fig. 2i). Though not present at E15, GFAPδ marks a population of bipolar cells in the VZ at E18, but more clearly at P0 (Fig. 2A,B,C). These cells extend their processes towards the lateral ventricle and stretch towards the pia, indicative of radial glial cells. Pan-GFAP immunoreactivity displays the same pattern as GFAPδ in the VZ, but from an earlier developmental stage onward (Fig. 2D,E,F). Radial glia cells are also present in the MPall. The GFAP expression pattern observed here mimics that seen in the VZ. Again, pan-GFAP immunoreactivity is detected at E12, and intensifies as development progresses. By E18, the majority of cells expressing GFAP are located in the fimbria (Fig. 2ii) or the dentate gyrus (DG; Fig. 2ii). Though not present at E12 (Fig. 2G), GFAPδ is strongly expressed in the DG E18 and P0 (Fig. 2H,I). GFAPδ marks a cluster of bushy cells with thick, stubby processes in the DG at E18 and P0. Pan-GFAP immunoreactivity marks relatively the same population of cells as GFAPδ in the DG, but from an earlier developmental stage (Fig. 2J,K,L).

### GFAPδ is Expressed by Embryonic Progenitors

GFAPδ and pan-GFAP mark specific populations of cells in the VZ, MPall, and along the pial surface during development. Cells that express both pan-GFAP and GFAPδ in the VZ have a bipolar morphology and are hypothesized to be radial glial cells. In order to investigate whether GFAPδ is indeed expressed in radial glia or in other cell types during development, a series of triple stainings was performed.

**Figure 3:**
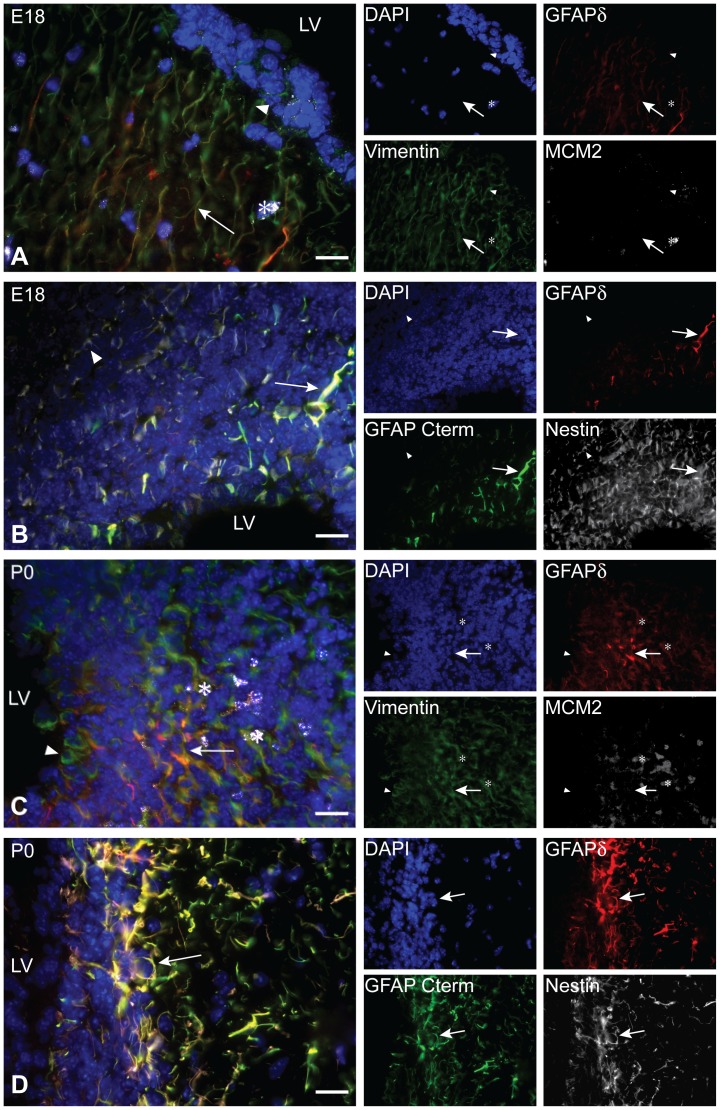
GFAPδ co-locolization profile in the developing SVZ. GFAPδ immunoreactivity always colocalizes with vimentin immunoreactivity (arrow, A), but vimentin marks a broader population of cells in the E18 VZ (arrow head, A). GFAPδ and MCM2 rarely colocalize (asterisk; A). GFAPδ, nestin, and GFAPα (marked by an antibody for the GFAP C-terminus) always colocalize (arrow, B). However, nestin expression is much more widespread than that of GFAP in the E18 VZ and SVZ (arrow head, B). Like in the E18 VZ, vimentin expression does overlap with GFAPδ expression (arrow, C), however vimentin expression marks a greater population of cells in the P0 VZ and SVZ (arrow head, C). MCM2 and GFAPδ can colocalize (asterisks, C). At P0, GFAPδ expression is broader in the VZ and SVZ than observed at E18. At P0, GFAPδ now encompasses all the GFAPα and nestin immunoreactivity (arrow, D). Abbreviation: LV: lateral ventricle. Scale bars = 20 *µ*m.

At E18, GFAPδ always colocalized with vimentin, a radial glia marker. However, all cells that expressed vimentin did not necessarily co-express GFAPδ (Fig. 3A, Sup. 1A). Those cells that expressed vimentin and GFAPδ were bipolar in their morphology. Focusing around the LV, both GFAPδ and vimentin could be found in the VZ and the SVZ, indicating that GFAPδ is expressed by both radial glia and basal progenitors. As these cell types divide, GFAPδ colocalization with a division marker was investigated. GFAPδ and/or vimentin positive cells were rarely seen to colocalize with minichromosome maintenance complex component 2 (MCM2), a marker for the initiation of cell replication (Fig. 3A, Sup. 1A). In the E18 VZ and SVZ, GFAPδ immunoreactivity was always seen to colocalize with GFAP C-terminus immunoreactivity (Fig. 3B, Sup. 1C). The GFAP C-terminus antibody marks GFAPα specifically in mouse. Notably, all GFAPδ positive cells were also positive for the stem cell marker nestin. However, nestin expression is far more widespread at E18 in the VZ and SVZ than that of GFAPδ (Fig. 3B, Sup. 1C).

The expression profile of the VZ at P0 mirrored that seen at E18. Again, all cells that expressed GFAPδ also expressed vimentin, but not necessarily the other way around. It was also rare to see a colocalization of MCM2 and GFAPδ (Fig. 3C, Sup. 1E). GFAPα immunoreactivity (as marked by the GFAP C-terminus antibody) is always seen to colocalize with GFAPδ immunoreactivity. Notably, nestin immunoreactivity has changed from P0 and now completely overlaps with that of GFAPδ (Fig. 3D, Sup. 1G). P0 marks the beginning of radial glia’s transformation into astrocytes as well as the start of the peak of astrogenesis. Here, cells that express GFAPδ are losing their bipolar phenotype while increasing the thickness and number of their processes.

**Figure 4:**
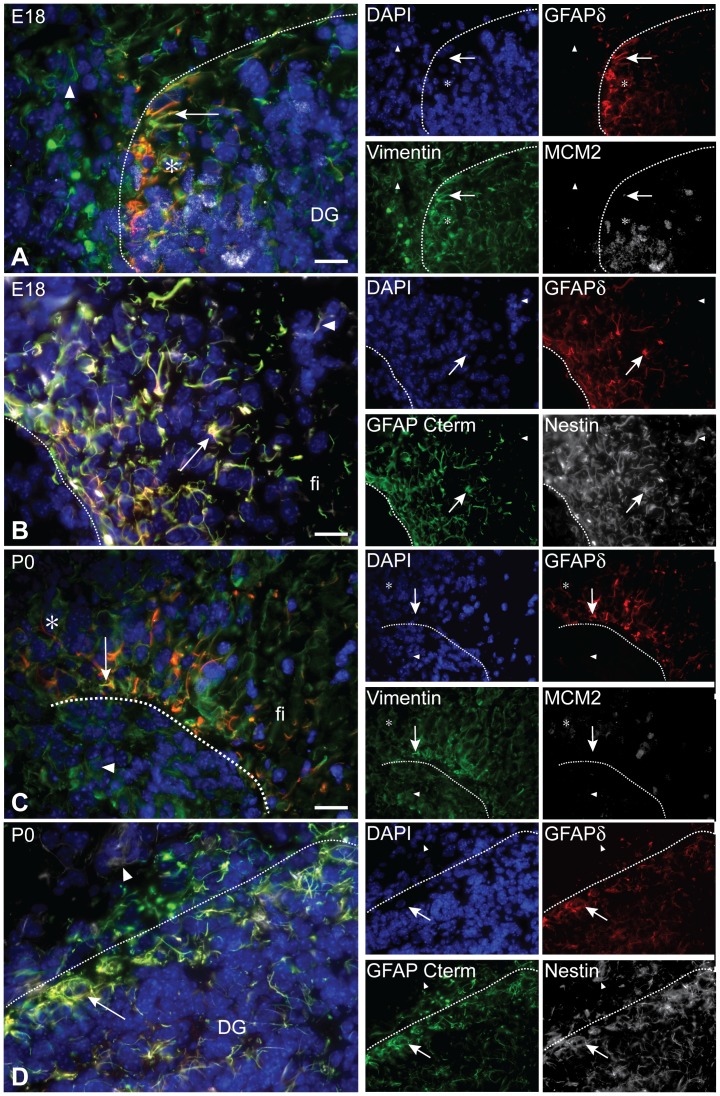
GFAPδ co-locolization profile in the developing hippocampus. Vimentin expression is more widespread than that of GFAPδ in E18 MPall (arrow head, A). However, GFAPδ commonly colocalizes with vimentin (arrow, A). GFAPδ cells rarely colocalize with the cell division marker MCM2 (asterisk, A). GFAPδ marks a distinct population of cells within the E18 DG (dashed line, A). GFAPδ, GFAPα, and nestin completely overlap in the developing DG and fimbria (arrow, B), however nestin marks a broader population of cells (arrow head, B). At E18, GFAPδ also marks a distinct population of cells within the developing fimbria (dashed line, B). This immunohistological pattern seen at E18 also carries through to the P0 hippocampus. Vimentin expression is more widespread than GFAPδ expression (arrow head, C), though there are double-positive populations as well (arrow, C) and MCM2 hardly colocalizes with GFAPδ (asterisk, C). However MCM2 expression is only seen in those distinct areas with GFAPδ immunoreactivity (dashed line, C). GFAPδ, GFAPα, and nestin mostly overlap in the P0 hippocampus (arrow, D), but nestin expression is far more widespread (arrow head, D). Again, GFAPδ immunoreactivity remains within the DG (dashed line, D). Abbreviations: DG: dentate gyrus, fi: fimbria. Scale bars = 20 *µ*m.

In the developing E18 hippocampus, GFAPδ, like in the VZ, always colocalizes with vimentin. However here in the hippocampus, vimentin marks a much broader population of cells than GFAPδ. The amount of cells expressing both GFAPδ and MCM2 is also very low (Fig. 4A, Sup. 1B). In the DG, MCM2 marks a small population of actively dividing cells. Few of these MCM2 positive cells are clearly GFAPδ positive (Fig. 4A, Sup. 1B). As expected, the expression of GFAPδ and GFAPα entirely overlaps. GFAPδ and GFAPα mark a very specific population of cells only located within the DG and fimbria (Fig. 4B). Nestin also completely colocalizes with GFAPδ in both the DG and the fimbria (Fig. 4B, Sup. 1D). At P0, the same basic expression pattern seen at E18 continues. In the DG and fimbria, most vimentin positive cells also express GFAPδ. Whereas in the surrounding hippocampal formation, there is no GFAPδ expression and, consequently, all vimentin positive cells are GFAPδ negative (Fig. 4C, Sup. 1F). Nestin and GFAPα completely colocalize with GFAPδ in the P0 hippocampus (Fig. 4D, Sup. 1H).

### Gfapδ mRNA is Detectable from E12

**Figure 5:**
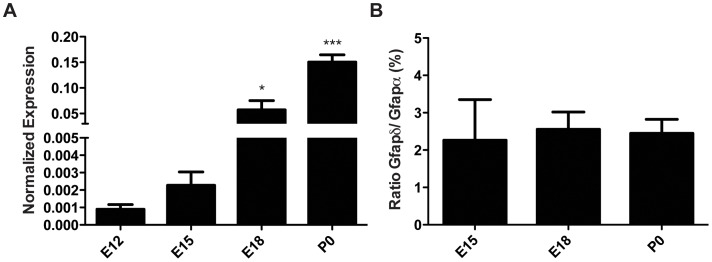
Gfap transcript expression in the developing mouse brain. Though the transcript levels of pan-Gfap increase throughout development (p = 0.001, Oneway ANOVA; A), the ratio between Gfapδ and Gfapα remains unchanged (B). All data is normalized to Gapdh and B-actin. Normalization procedures are described extensively in [43]. Gfapα and Gfapδ are detected with equal efficiencies. The same Rn threshold was used in all qPCRs. Ratios are calculated as Gfapδ/Gfapα x 100. Data is displayed as mean ± s.e.m.

In order to obtain more detailed quantitative information on Gfap isoform expression level during development, real time quantitative PCR (qPCR) was performed. Whole brains of E12 through P0 mice were subjected to RNA isolation and qPCR analysis. The samples were first investigated for pan-Gfap expression. Transcript levels of most Gfap isoforms, as discerned with pan-Gfap primers, were detectable from E12 and progressively increased during development (Fig. 5A). There was a 150- fold increase of pan-Gfap expression from E12 to P0. Subsequently, the transcription levels of Gfapδ and the canonical isoform Gfapα were then determined.

All samples expressed Gfapα throughout every stage studied. Moreover, as development progressed, the transcript levels of Gfapα increased. Although GFAPδ protein was unable to be detected at E12 and E15, the Gfapδ transcript was detected. At E12, some (3 out of 5) samples had detectable Gfapδ transcript levels, albeit at low level. At E15, all samples displayed detectable levels of Gfapδ. The ratio between Gfapδ and Gfapα expression was investigated from E15 to P0, as all samples in these stages expressed both Gfapδ and Gfapα. The Gfapδ/Gfapα ratio did not significantly change at any point in development (Fig. 5B). This finding clearly shows that the total brain Gfapδ/Gfapα transcript ratio remains stable throughout both neurogenesis and gliogenesis.

### GFAPδ is Expressed by All Cells that Express pan-GFAP in the Adolescent Mouse Brain

In order to track GFAPδ positive cells through later development, brains from early postnatal ages into adolescence were profiled using a series of antibodies. Pan-GFAP immunoreactivity is seen throughout the entire adolescent mouse brain. Though most notable in the astrocytes of the DG and SVZ, GFAP expression is also observed in astrocytes of the cortex. Surprisingly, all astrocytes that were marked by the pan-GFAP antibody, also expressed GFAPδ. The expression patterns of GFAPδ and pan-GFAP immunoreactivity were highly similar, but differed among brain regions. Both GFAPδ and pan-GFAP displayed strong immunoreactivity in subcortical areas. GFAPδ displayed a weaker immunoreactivity in regions such as the cortex while pan-GFAP immunoreactivity was more readily detectable.

**Figure 6:**
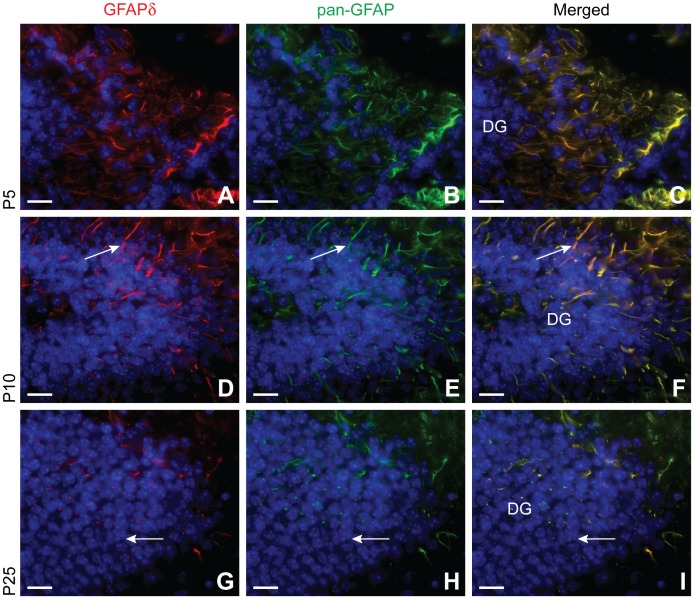
GFAP expression in the young and adolescent hippocampus. GFAP expression is still increasing until P5 (A–C), then it begins to decrease until it reaches a plateau around P25 (G–I). There is also a dramatic morphological change around this time, GFAP expressing cells begin to organize in a more parallel fashion by extending long fibers across the DG (arrow, D–F). These fibers continue to lengthen, as seen by the fine, punctate staining at P25 (arrow, G–I). GFAPδ expression mimics that of other GFAP isoforms throughout all timepoints. Abbreviation: DG: dentate gyrus. Scale bars = 20 µm.

At P5, many cells in the DG show pan-GFAP immunoreactivity (Fig. 6A,B,C). These cells have a relatively bushy morphology and are clustered closely together. As the animal matures, these pan-GFAP positive cells seem to extend their processes in a more organized fashion, resulting in a striking bipolar morphology around P10 (Fig. 6D,E,F) and long-range fibers at P25 (Fig. 6G,H,I). This shift in morphology seems to coincide with a decrease in pan-GFAP immunoreactivity. All cells that express pan-GFAP immunoreactivity also express GFAPδ. The staining pattern of GFAPδ completely overlaps with pan-GFAP immunoreactivity. In fact, the DG displays ubiquitous GFAPδ expression in the same cell compartments as other GFAP isoforms.

**Figure 7:**
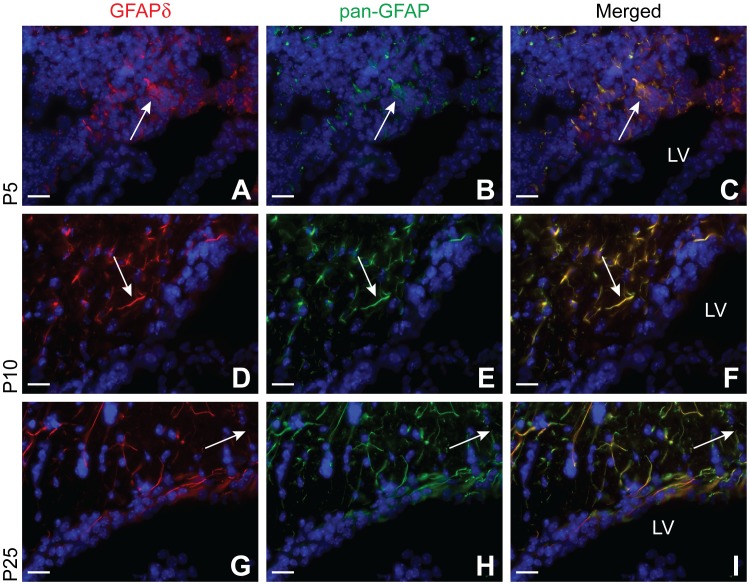
GFAP expression in the young and adolescent subventricular zone. At P5, GFAP expressing cells still resemble bipolar radial glia cells (arrow, A–C). From P5 (A–C) to P10 (D–F), both GFAPδ and GFAP isoform expression increases as denoted by the thick fibers transversing the SVZ (arrow). At P25, there is a slight reduction in GFAP expression, illustrated by the punctate staining in the SVZ (G–I). From P10 (D–F) to P25 (G–I), GFAP isoform expression expands into more distal processes (arrow) of the cell. Abbreviation: LV: lateral ventricle. Scale bars = 20 µm.

Around P5 in the SVZ, the cells that show pan-GFAP immunoreactivity, like in the DG, are heavily clustered together. These cells also have a bipolar morphology (Fig. 7A,B,C), as already clearly seen by E18. From P10 to P25, these processes begin to grow and infiltrate the surrounding parenchyma. From P10 (Fig. 7D,E,F), but most notable at P25, the processes extend and thin out, as denoted by the punctate staining patterns in the SVZ and surrounding parenchyma (Fig. 7G,H,I).

**Figure 8:**
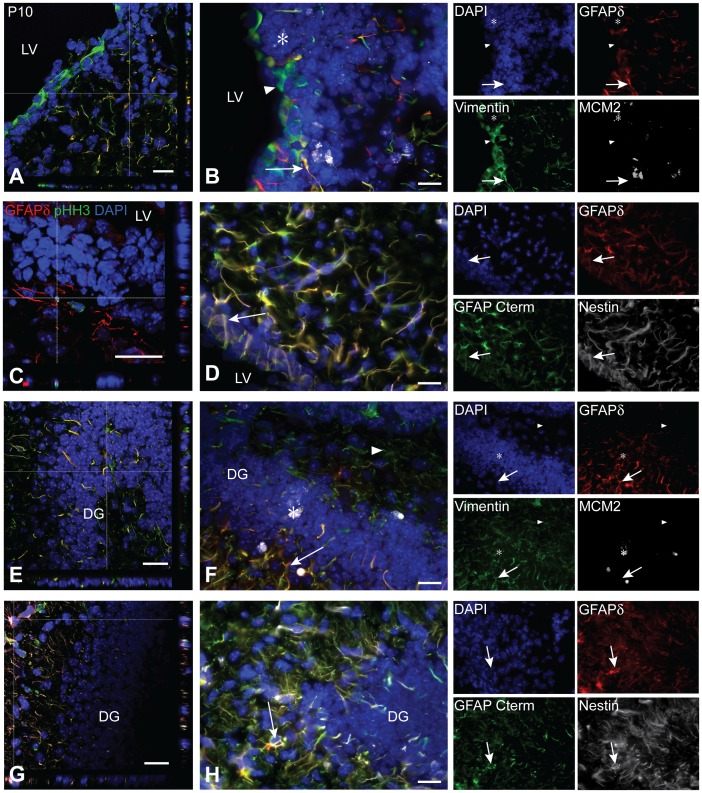
GFAPδ co-localization profile in the adolescent subventricular zone and hippocampus. GFAPδ and vimentin expression can overlap in the P10 SVZ (A; arrow, B), however there are also populations of GFAPδ, vimentin negative and GFAPδ negative, vimentin positive cells (A; arrow head, B). GFAPδ cells rarely divide as seen by the lack of colocalization with cell division marker MCM2 (A; asterisk, B). GFAPδ sometimes colocalizes with the cell division marker pHH3 (C). Nestin, GFAPα (as marked by the GFAP C-terminus antibody), and GFAPδ always colocalize (D). Some of these cells show the typical morphology of B1 quiescent astrocytes (arrow, D). Vimentin immunoreactivity is more widespread than that of GFAPδ in the P10 hippocampus (E). There are populations of vimentin positive, GFAPδ negative cells (arrow head, F), as well as vimentin and GFAPδ double positive cells (arrow, F). GFAPδ positive cells rarely divide (E; asterisk, F). As in the SVZ, GFAPα, GFAPδ, and nestin always colocalize in the DG (G; arrow, H). Abbreviations: LV: lateral ventricle, DG: dentate gyrus. Scale bars = 20 µm.

In the P10 SVZ, vimentin expression marks a population of ependymal cells lining the lateral ventricle as well as a restricted population of astrocytes in the SVZ parenchyma. GFAPδ immunoreactivity and vimentin expression can overlap but there are also exclusive vimentin positive and GFAPδ positive cell populations present. Just as in development, the colocalization between MCM2 and GFAPδ is rare (Fig. 8A,B). As colocalization of GFAPδ and MCM2 was quite rare, other proliferation markers were also profiled. However even using a broader marker of proliferation, such as phosphohistone-H3 (pHH3), still resulted in limited colocalization with GFAPδ (Fig. 8C). Notably, both MCM2 and pHH3 positive cells were located within classical neurogenic niches like the SVZ and SGZ. Nestin, GFAPα, and GFAPδ are found to reliably colocalize within the same cells (Fig. 8D). In the P10 DG, vimentin marks a greater population of cells than GFAPδ. That said, all GFAPδ positive cells are also vimentin positive. GFAPδ immunoreactivity hardly overlaps with that of MCM2 (Fig. 8E,F). As in the P10 SVZ, GFAPα, GFAPδ, and nestin expression completely overlap (Fig. 8G,H).

### GFAPδ Displays a Similar Profile *in vitro* as it does *in vivo*

**Figure 9:**
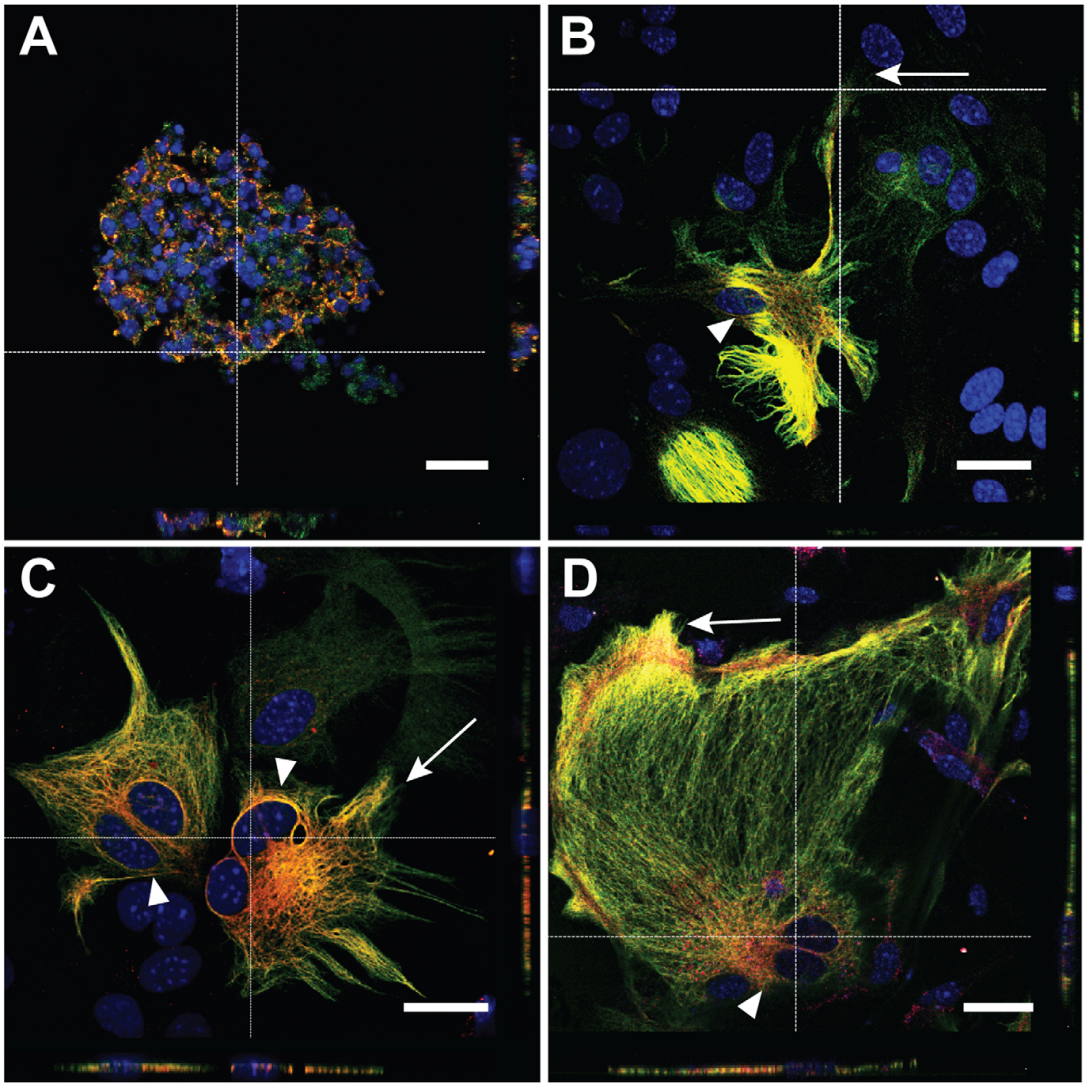
*In vitro* expression of GFAPδ. Primary neurospheres (A) and astrocytes (B–D) express both GFAPδ and GFAPα. GFAPα, not GFAPδ, can be clearly seen in the outer edges of the IF network (arrow, B–C). GFAPδ is commonly found to surround the nucleus and fill the soma (arrow heads, C–D), and can, in some instances, be seen in the outer edges of the IF network (arrow, D). To determine whether a dye-swap had an effect on localization detection, a different secondary antibody conjugated to a different fluorphore was used. This dye-swap rendered the same results (D, image is recolored for presentation purposes). B–D are recorded using identical settings. GFAPδ is shown in red. GFAPα, as marked by an antibody against the GFAP C-terminus, is shown in green. DAPI is shown in blue. Scale bars = 20 µm.

Primary astrocyte and primary neurosphere cultures from P0–P3 mice were made to investigate mouse GFAPδ more closely *in vitro*. Firstly, cultures were assessed by qPCR to determine the Gfapδ/Gfapα transcript ratio. Both neurosphere and primary astrocyte cultures expressed Gfapδ and Gfapα transcripts. Moreover, the Gfapδ/Gfapα transcript ratio did not significantly differ between neurosphere and astrocyte cultures (neurospheres: 9.378±0.4725; astrocytes: 7.615±1.715; unpaired t test p = 0.2498). Interestingly, the Gfapδ/Gfapα ratio shifts *in vitro* (7.6) and *in vivo* (2.5). Both primary neurosphere cultures (Fig. 9A) and primary astrocytes (Fig. 9B,C,D) expressed GFAPδ. Primary astrocyte cultures showed a complex phenotype, where GFAPα was commonly observed alone in the tips of the IF network (Fig. 9B,C). GFAPδ expression was usually localized around the nucleus and in the soma (Fig. 9B,C,D). Performing a dye-swap experiment, where the secondary fluorphores were switched, resulted in the same finding (Fig. 9D). However, the GFAPδ distribution throughout the IF network was variable, and could even be observed in the edges of the IF network (Fig. 9D).

## Discussion

From the investigation of GFAPδ during mouse development, it appears that if GFAPδ is detectable, it is not limited to neurogenic cells. GFAPδ immunoreactivity is first seen at E18. Here, GFAPδ is mainly localized within radial glia of the VZ and MPall. As the animal matures, GFAPδ positive cells begin to lose their bipolar morphology and shift towards a more star-like, mature phenotype. This shift is most evident from P5 to P10 in both the SVZ and hippocampus. At P25, GFAPδ positive cells show complex branching and extended processes, indicative of mature astrocytes. GFAPδ positive cells are also present in the classical neurogenic niches, reflecting the different types of SVZ astrocytes, including quiescent neurogenic astrocytes.

Throughout all timepoints studied, the expression of vimentin and nestin are mostly more widespread than that of GFAPδ. In early stages of development (E18 and P0) the colocalization of GFAPδ with vimentin and nestin indicates that GFAPδ is expressed by both radial glia and undifferentiated precursors [44]–[46]. Interestingly, GFAPδ is not homogenously present within these niches, which may indicate that GFAPδ marks a subpopulation at E18. More cells acquire detectable GFAPδ expression at P0. Taken together, these data could indicate that a subpopulation of radial glia cells is maturing, thereby amassing GFAP expression [47]–[49]. This process commences around E18, coinciding with the start of astrogenesis, and advances through P0 - where more colocalization between GFAPδ and vimentin can be seen, due to the greater expression of GFAPδ at this timepoint. In addition to radial glia, GFAPδ is also observed around the developing anterior commissure. These astrocytes excrete growth factors and forming physical barriers, allowing for the proper formation of commissures and, in turn, proper development of neural circuitry [50], [51]. Strong GFAPδ expression is also observed from E18 demarcating the supragranular and fimbrial bundles. These structures are crucial for hippocampal morphogenesis. Without the supragranular bundle, the DG is unable to fully form [52]. The presence of GFAPδ in both radial glia and other astrocyte populations suggest that GFAPδ may play a critical role in general astrocyte biology and is an integral part of the GFAP intermediate filament cytoskeleton.

The maturation of GFAPδ positive cells coincides with differential marker expression. From P10, GFAPδ marker co-expression shifts towards a more mature phenotype. Here, GFAPδ positive cells seem to have lost most of their vimentin expression. This observation fits well with the GFAPδ/vimentin pattern seen in development. Moreover, the reduced colocalization between vimentin and GFAPδ in the SVZ indicates that GFAPδ is not expressed in ependymal cells; as vimentin is commonly reported as an ependymal marker in the mature SVZ [45], [48], [53]. Cells expressing GFAPδ can divide, as marked by MCM2 and pHH3 co-expression. However, they do so rarely and only within the SVZ and SGZ. At P10, nestin expression is more restricted than at E18 and P0, and completely overlaps with GFAPδ immunoreactivity. This refined nestin expression along with its co-expression with GFAPδ indicates that neurogenic astrocytes in the SVZ and SGZ express GFAPδ [54], [55].

Contrary to vimentin and nestin expression, GFAPα and GFAPδ always colocalize from E18 onwards. This finding is unsurprising as GFAPδ, unlike the canonical isoform GFAPα, is unable to form a functional IF network by itself [13], [14]. This observation is in accordance to what has been seen in the adult mouse brain [10], where GFAPδ and other GFAP isoforms such as GFAPα mostly colocalize within the same cell, whether that cell has a neurogenic phenotype or not. Notably, pan-GFAP immunoreactivity is observed 6 days prior to the appearance of GFAPδ. Pan-GFAP immunoreactivity was observed in radial glia cells, analogous to previous reports [20]. The discrepancy between the appearance of pan-GFAP and GFAPδ immunoreactivity could be attributed to the lower abundance of GFAPδ transcript level (7.9% of Gfapα in the adult mouse brain [10]) or to a delay in GFAPδ translation.

Even though GFAPδ was first seen at E18 using immunohistochemistry, Gfapδ transcripts were first seen much earlier, around E12 to E15. Not all samples express Gfapδ at E12 (3 out of 5 samples) and those that do, express very low levels of Gfapδ. The discrepancy between Gfap transcript and GFAP protein detection has been described before *in vivo* and *in *vitro [56]–[61]. Most strikingly, when Zhou and colleagues (2000) studied the stratum radiatum of the CA1, a glial dense region in the hippocampus, they found that there was much more expression of the Gfap transcript (74% of all cells) than of the GFAP protein (1.5% of all cells). Furthermore, there are reports that GFAP protein expression is preceded by Gfap transcript expression [57], [58]. Although this phenomenon could be attributed to the lack of sensitivity of a given GFAP antibody, it is more likely due to a translational mechanism [60]. Interestingly, an altered translational mechanism retarding GFAP protein production also implies a distinctive role for Gfap mRNA, itself.

If the Gfapδ transcript would have a specific neurogenic or astrogenic role, the Gfapδ/Gfapα ratio would be expected to shift throughout different developmental stages. However, there is no significant change in the Gfapδ/Gfapα ratio from E15 to P0. The Gfapδ/Gfapα transcript ratio is higher *in vitro* than *in *vivo. This finding may be due to the stress of an *in vitro* environment to the cell, causing the cell to change the fundamental composition of its IF network. That said however, the Gfapδ/Gfapα transcript ratio also does not differ between primary astrocytes and primary neural stem cell cultures. These data are in accordance with previous findings from our lab, which show that the Gfapδ/Gfapα transcript ratio remains stable throughout adult neurogenic and non-neurogenic mouse brain regions, even though the amount of transcript expression is variable [10].

Taken together, these results highlight the divergence between human and mouse GFAPδ. In the adult human SVZ, there is a higher Gfapδ/Gfapα transcript ratio when compared to a non-neurogenic region [14]. Unlike the developing mouse brain, human GFAPδ (hGFAPδ) simultaneously emerges with other GFAP isoforms at 13 weeks of gestation in the developing human VZ. GFAPδ is present in radial glia in both the developing human and mouse. However, hGFAPδ stays confined to the VZ and the SVZ during development. To that end, pan-GFAP immunoreactivity is far more widespread than that of hGFAPδ, which is in stark contrast to the situation in mouse where pan-GFAP immunoreactivity always coincides with that of GFAPδ. Marker co-expression also differs between species. Human vimentin, nestin, and hGFAPδ always colocalize in development [16], whereas the vimentin and nestin patterns in the embryonic mouse are far more widespread. This finding also supports previous evidence that primate radial glia undergo the transition from vimentin to GFAP expression earlier than rodent counterparts [62]. Most hGFAPδ positive cells co-express a proliferation marker [16]. Proliferation markers were extensively investigated in this study, however the presence of a dividing cell expressing GFAPδ was a rarity. GFAPδ seems to demarcate a more specialized cell-type in human than mouse. hGFAPδ positive cells show a more defined, homogenous phenotype during development.

The current study set out to explore the involvement of GFAPδ during mouse developmental neurogenesis. The results of this study indicate that mouse GFAPδ and hGFAPδ have differential expression patterns. Moreover, mouse GFAPδ is only detectable well after the peak of embryonic neurogenesis, around the commencement of astrogenesis. During adolescence, GFAPδ does not demarcate a specific population of neural stem cells in the mouse brain as it does in the human brain. *In vitro*, GFAPδ is present in both multipotent, self-renewing neural stem cells as well as astrocytes. The Gfapδ/Gfapα transcript ratio neither shifts during development nor in primary astrocyte and neural stem cell cultures. During development, GFAPδ is expressed by a population of radial glia cells, which seem to be acquiring a more mature phenotype. However, it is also expressed by astrocytes involved in commissure formation. In the adolescent hippocampal formation, GFAPδ is expressed in the neurogenic SGZ but also in the non-neurogenic fimbria. Taken together these observations indicate that though GFAPδ is not a specific neural stem cell marker in the developing mouse brain, it may be an integral part of the intermediate filament network of all developing and mature astrocytes.

## Supporting Information

Figure S1 **Confocal analysis of GFAPδ colocolization in the developing VZ and MPall.** In the E18 VZ and DG, GFAPδ always colocalizes with vimentin and rarely with MCM2. Here, vimentin expression is far more widespread than that of GFAPδ (A–B). At this developmental stage, GFAPδ always colocalizes with GFAPα and nestin. However like vimentin expression, nestin marks a broader range of cells than GFAPδ (C–D). At P0, the separation of vimentin and GFAPδ expression becomes more evident, where vimentin is commonly seen in GFAPδ ependymal cells with the VZ (E). Again, colocalization between GFAPδ and MCM2 is rare in both the VZ and the DG (E–F). Nestin expression has also transitioned at P0. Now, all cells that express nestin also express both GFAPδ and GFAPα within the VZ (G). However, nestin still marks a larger population of cells in the hippocampus than GFAPδ (H). Abbreviations: LV: lateral ventricle, DG: dentate gyrus. Scale bars = 20 µm.
(TIF)
