# Supplementary figures and images for: GFAPδ Expression in Glia of the Developmental and Adolescent Mouse Brain
Source: PLoS One. 2012 Dec 21;7(12):e52659. doi: 10.1371/journal.pone.0052659 (PMC3528700; doi:10.1371/journal.pone.0052659)

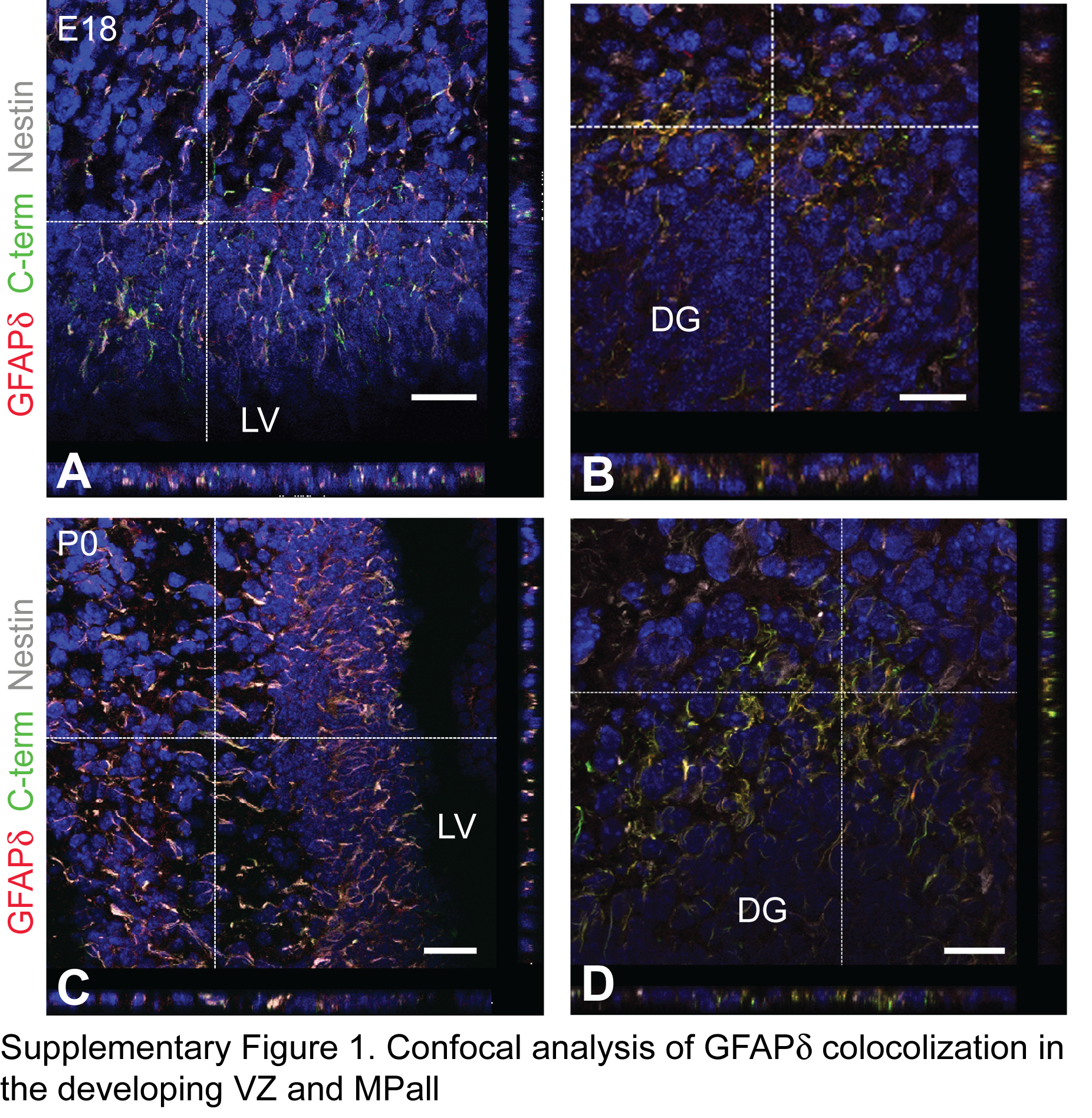

Supplement: Figure S1 — Confocal analysis of GFAPδ colocolization in the developing VZ and MPall. In the E18 VZ and DG, GFAPδ always colocalizes with vimentin and rarely with MCM2. Here, vimentin expression is far more widespread than that of GFAPδ (A–B). At this developmental stage, GFAPδ always colocalizes with GFAPα and nestin. However like vimentin expression, nestin marks a broader range of cells than GFAPδ (C–D). At P0, the separation of vimentin and GFAPδ expression becomes more evident, where vimentin is commonly seen in GFAPδ ependymal cells with the VZ (E). Again, colocalization between GFAPδ and MCM2 is rare in both the VZ and the DG (E–F). Nestin expression has also transitioned at P0. Now, all cells that express nestin also express both GFAPδ and GFAPα within the VZ (G). However, nestin still marks a larger population of cells in the hippocampus than GFAPδ (H). Abbreviations: LV: lateral ventricle, DG: dentate gyrus. Scale bars = 20 µm. (TIF) [file pone.0052659.s001.tif]
